# High Defect Formation Energies Limit Ionic Conductivity in Na_3_Ga_2_Li_3_F_12_ and Na_3_Al_2_Li_3_F_12_

**DOI:** 10.1021/acs.chemmater.6c01542

**Published:** 2026-10-23

**Authors:** Michael J. Brady, Hauke Glück, Jessica L. Andrews, Erica Truong, Alexander G. Squires, Ifeoluwa P. Oyekunle, Rachel C. Monger, Wolfgang G. Zeier, Yan-Yan Hu, David O. Scanlon, Benjamin J. Morgan, Brent C. Melot

**Affiliations:** † Department of Chemistry, 5116 University of Southern California, Los Angeles, California 90089, United States; ‡ Institute for Inorganic and Analytical Chemistry, 9185 University of Münster, Corrensstrasse 30, Münster D-48149, Germany; § Department of Chemistry and Biochemistry, 7823 Florida State University, Tallahassee, Florida 32306, United States; ∥ School of Chemistry, 4919 University of Birmingham, Birmingham B15 2TT, U.K.; ⊥ The Faraday Institution, Quad One, Harwell Science and Innovation Campus, Didcot OX11 0RA, U.K.; # Department of Chemical Engineering and Materials Science, 5116 University of Southern California, Los Angeles, California 90089, United States; ∇ Institute of Energy Materials and Devices (IMD), IMD-4: Helmholtz-Institut Münster, Forschungszentrum Jülich, Corrensstraße 46, Münster D-48149, Germany; ○ Center for Interdisciplinary Magnetic Resonance, National High Magnetic Field Laboratory, Tallahassee, Florida 32306, United States; ◆ The Faraday Institution, Quad One, Harwell Science and Innovation Campus, Didcot OX11 0RA, U.K.; ¶ Department of Chemistry, 1555 University of Bath, Claverton Down, Bath BA2 7AY, U.K.; ▶ Departments of Chemistry and Materials, 14787 Binghamton University, Binghamton, New York 13850-6000, United States

## Abstract

Lithium-conducting fluoride garnets exhibit ionic conductivities of ∼10^–10^ S cm^–1^, far below those of their oxide counterparts. Here, we investigate the origin of this poor transport in Na_3_Ga_2_Li_3_F_12_ and Na_3_Al_2_Li_3_F_12_ through a combination of structural characterization and first-principles calculations. Both compounds are highly ordered, with no significant antisite disorder, and exhibit intrinsic ionic conductivities of ∼2 × 10^–11^ S cm^–1^ at 100 °C. Nudged elastic band calculations reveal low Li-ion migration barriers (0.16 to 0.29 eV), indicating that the fluoride garnet framework does not inherently restrict Li-ion diffusion. However, the formation energies for lithium Frenkel and Schottky defect pairs are high (1.9 to 2.4 eV), indicating that the intrinsic concentrations of mobile Li-ion defects are negligibly small. These results establish that the low conductivity of stoichiometric fluoride garnets arises from high defect-formation energies, rather than high migration barriers.

## Graphical abstract

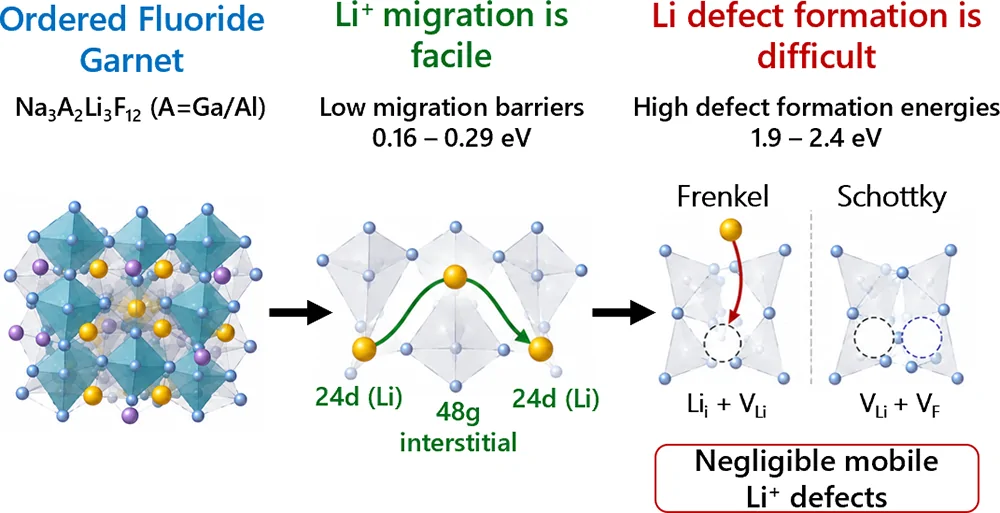

## Introduction

All-solid-state batteries require solid electrolytes that combine high Li-ion conductivity with electrochemical stability against metallic lithium.^1^ The oxide garnets, exemplified by Li_7_La_3_Zr_2_O_12_ (LLZO), are among the few material families to achieve both, with room-temperature conductivities above 10^–3^ S cm^–1^ and a nearly 6 V stability window.^2,3^ Oxide garnets, however, are susceptible to lithium dendrite penetration, which has been attributed to electronic conductivity arising from band gap narrowing at grain boundaries.^4,5^ Materials with wider bulk band gaps may be more resilient against this degradation pathway, which has motivated interest in fluoride-based garnet analogs, as the greater electronegativity of fluorine is expected to give wider band gaps than in the corresponding oxides.^6–8^

Fluoride garnets reported to date all adopt the stoichiometric Li_3_ *A*_2_ *C*_3_F_12_ composition and exhibit very low ionic conductivities of ∼10^–10^ S cm^–1^.^9^ In oxide garnets, high conductivity requires a “stuffed” composition in which the lithium content exceeds this stoichiometric Li_3_ level, combined with sufficient lithium disorder to enable cooperative transport.^2^ Stoichiometric oxide garnets such as Li_3_Nd_3_Te_2_O_12_ show negligible conductivity at room temperature,^10^ due to strong lithium ordering. The poor performance of the fluoride garnets is therefore consistent with their stoichiometric lithium content, but the origin of the low conductivity has not been investigated. In particular, it remains unclear whether a fluorine anion framework intrinsically restricts Li-ion migration, or whether a fluoride garnet framework can in principle support fast transport but is limited by some other factor, for instance, an inability to generate sufficient mobile Li-ion defects at accessible temperatures.

Here, we examine this question through a combined experimental and computational study of Na_3_Ga_2_Li_3_F_12_ and Na_3_Al_2_Li_3_F_12_. Synchrotron X-ray diffraction, neutron pair distribution function analysis, and ^71^Ga/^27^Al MAS NMR show that both compounds are highly ordered with no significant antisite disorder. Electrochemical impedance spectroscopy under controlled atmospheres reveals intrinsic ionic conductivities of ∼2 × 10^–11^ S cm^–1^ at 100 °C. First-principles nudged elastic band calculations, however, show that the Li-ion migration barriers are low (0.16 to 0.29 eV), indicating that the fluoride garnet framework does not inherently restrict Li-ion diffusion. Calculated formation energies for lithium Frenkel and Schottky defect pairs, though, are high (1.9 eV to 2.4 eV), indicating that the intrinsic concentrations of mobile Li-ion defects are negligibly small and identifying defect formation as the primary bottleneck limiting ionic conductivity in these materials.

## Results and Discussion

To identify the origin of low ionic conductivity in fluoride garnets, we first characterize the average and local structure of Na_3_Ga_2_Li_3_F_12_ and Na_3_Al_2_Li_3_F_12_ and measure their transport properties under controlled atmospheres, before turning to first-principles calculations to distinguish between migration-barrier and defect-concentration explanations.

Na_3_Ga_2_Li_3_F_12_ and Na_3_Al_2_Li_3_F_12_ were synthesized hydrothermally from binary fluorides dissolved in concentrated HF (see Experimental Methods for details). Both compounds crystallize in the garnet space group *Ia*3̅*d* (Figure 1), with Ga or Al on octahedral 16*a* sites, Li on tetrahedral 24*d* sites, and Na on 8-coordinate 24*c* sites. Rietveld refinements against synchrotron X-ray powder diffraction data confirm phase purity, with the resulting structural parameters listed in Tables S1–S2. The Ga-based phase is obtained phase-pure as prepared, whereas a minor AlF_3_ impurity (<5 wt %) is present in the aluminum analog (Figure 2). Scanning electron microscopy (SEM) images of Na_3_Ga_2_Li_3_F_12_ (Figure 2a inset) reveal uniform and well-faceted particles on the order of 5 μm, while Na_3_Al_2_Li_3_F_12_ (Figure 2b inset) shows much larger and less uniform particles.

**Figure 1:**
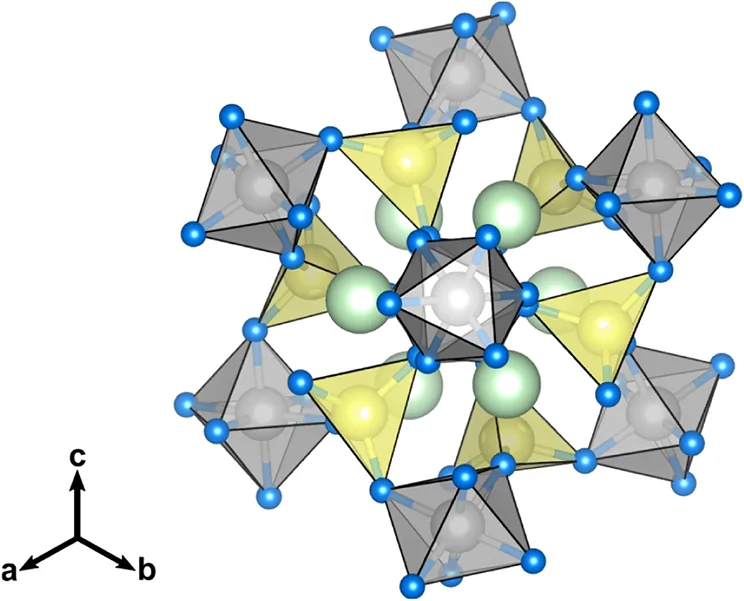
Garnet structure of Na_3_ *A*_2_Li_3_F_12_ (*A* = Ga, Al) viewed along the [111] body diagonal. The structure consists of a body-centered cubic packing of rods running along the body diagonals,^11,12^ each composed of alternating face-sharing octahedra (gray, Ga/Al) and trigonal prisms (not shown). Li tetrahedra (yellow) and Na 8-coordinate sites (green) occupy the inter-rod spaces. Fluorine atoms shown in blue. Crystal structure depicted using VESTA.^13^

**Figure 2:**
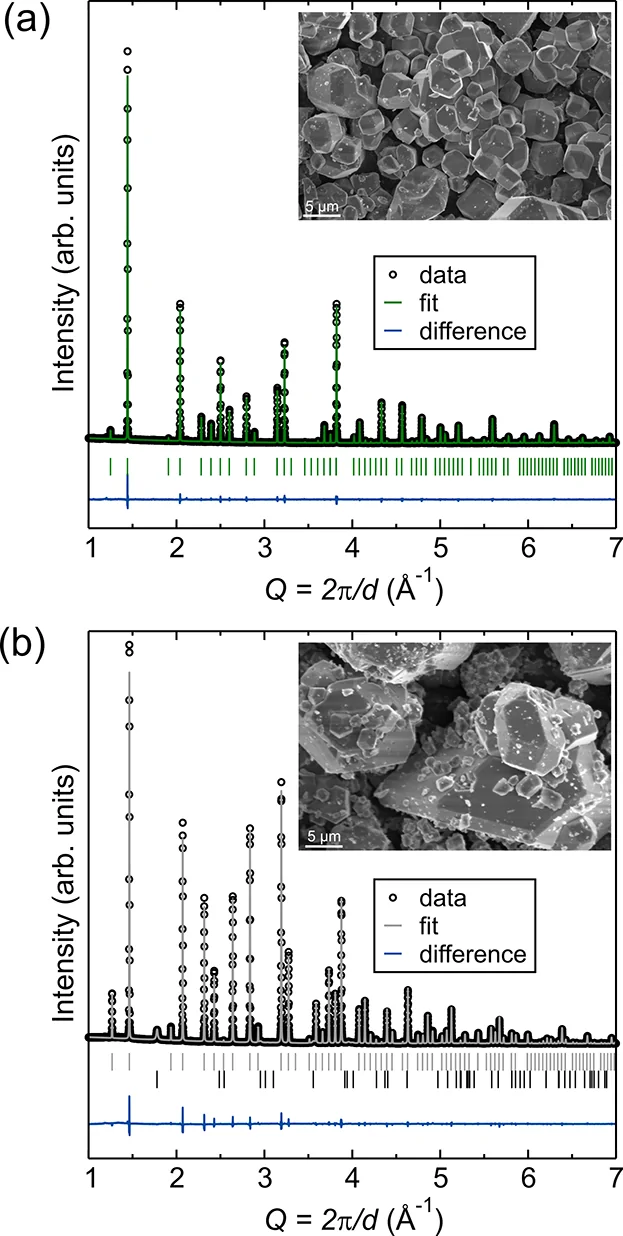
Rietveld refinements of (a) Na_3_Ga_2_Li_3_F_12_ (top, green) and (b) Na_3_Al_2_Li_3_F_12_ (bottom, gray). Colored tick marks indicate Bragg reflections of the garnet phase; black tick marks in (b) indicate the AlF_3_ impurity. Insets: SEM images of (a) Na_3_Ga_2_Li_3_F_12_ and (b) Na_3_Al_2_Li_3_F_12_ particles.

Electrochemical impedance spectroscopy was used to characterize the ionic conductivity of Na_3_Ga_2_Li_3_F_12_ and Na_3_Al_2_Li_3_F_12_. Pellets of polycrystalline powders (∼90% theoretical density) were formed using a uniaxial press, followed by cold-isostatic pressing and subsequent gold sputtering to create ion-blocking electrodes. No systematic study on the pellet density was performed; therefore, the roughly 90% dense pellets may underestimate the bulk conductivity due to porosity, contact between the particles, and unresolved grain-boundary resistance. The initial impedance spectra of the gallium phase under ambient conditions (Figure S2) showed an anomalously high room-temperature conductivity on the order of 10^–7^ S cm^–1^ that increased up to 75 °C, above which the data dropped into the noise floor. On cooling below this threshold, the high-conductivity semicircle reemerged, confirming the effect is reversible. Measurement under an argon atmosphere suppressed the low-frequency tail in the impedance spectra, suggesting a surface phenomenon.

To test whether this anomalous conductivity is atmosphere-dependent, impedance measurements were collected while alternating between humid and dry nitrogen. Figure 3a shows a large increase in apparent conductivity for Na_3_Ga_2_Li_3_F_12_ under humid conditions, while Na_3_Al_2_Li_3_F_12_ (Figure 3b) shows minimal change. The rapid reversibility of this effect when switching between humid and dry atmospheres, together with its suppression under inert Ar, suggests a surface-related process rather than a change in bulk transport. From the SEM images, characteristic particle dimensions of approximately 5 μm for Na_3_Ga_2_Li_3_F_12_ and approximately 10–20 μm for Na_3_Al_2_Li_3_F_12_ can be observed. Approximating the particles as nonporous spheres, the Ga powder therefore has an estimated external geometric surface area per unit solid volume roughly two to four times larger. This estimate is qualitative because particle shape, agglomeration, pellet porosity, and internal surface area are not captured, but is sufficiently different that the increased surface area could explain the different in sensitivity to moisture. An alternative explanation would be bulk Li^+^/H^+^ exchange with atmospheric moisture, as observed in Li-stuffed oxide garnets. In these Li-stuffed materials, excess lithium beyond the stoichiometric 24*d* occupancy provides a reservoir of readily extracted Li.^14^ In the stoichiometric fluoride garnets, however, all Li resides on structural 24*d* sites, and extracting it is expected to be energetically costly, making such exchange unlikely. Consistent with this, FTIR spectra (Figure S6) show no absorption features in the O–H stretching region near 3500 cm^–1^, and TGA (Figure S5) shows minimal weight loss below 200 °C, ruling out significant bulk moisture incorporation. The humidity-induced conductivity in Na_3_Ga_2_Li_3_F_12_ is therefore attributed to an extrinsic surface phenomenon.

**Figure 3:**
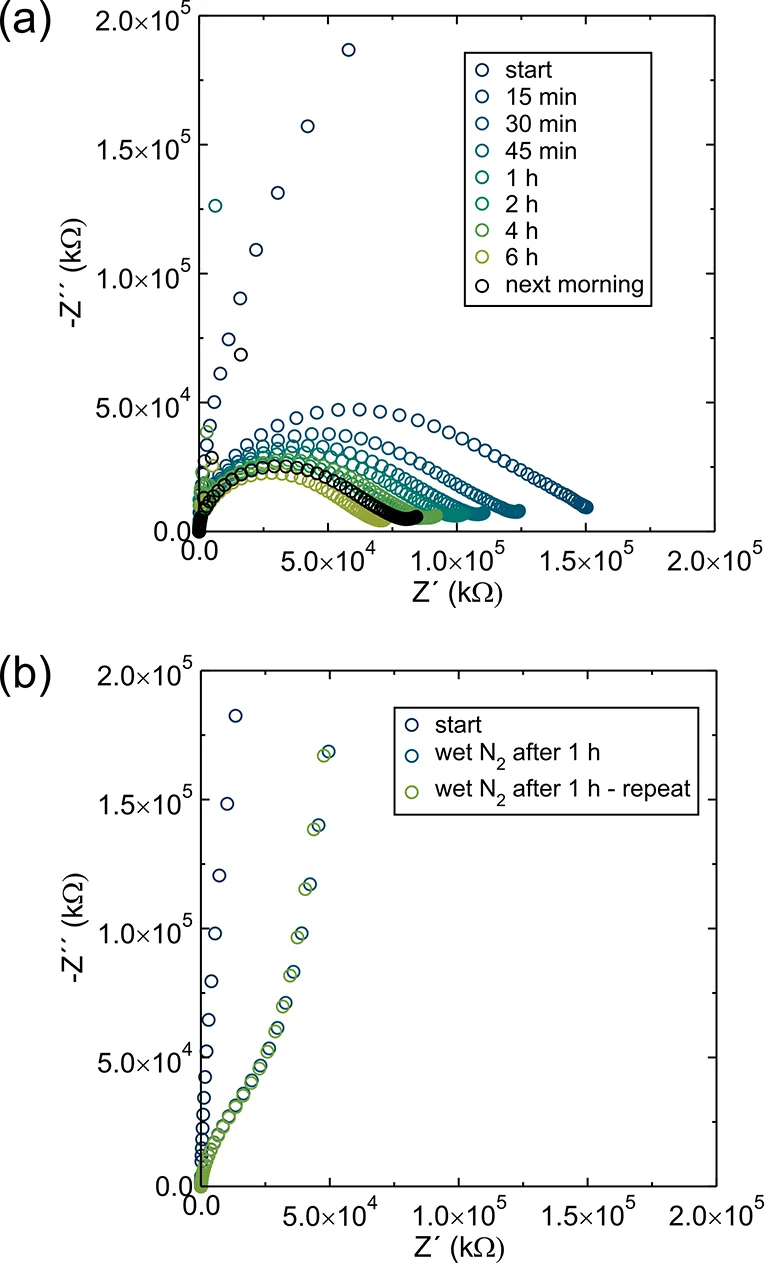
EIS Nyquist plots under humid nitrogen (generated by flowing dry N_2_ through saturated Ca­(NO_3_)_2_ solutions) for (a) Na_3_Ga_2_Li_3_F_12_, showing a progressive increase in apparent conductivity over several hours, and (b) Na_3_Al_2_Li_3_F_12_, which shows minimal change.

To isolate the intrinsic Li-ion conductivity from the surface contribution, we performed elevated-temperature measurements under inert atmosphere. Gold-coated pellets of each material were sandwiched between aluminum tabs and vacuum-sealed inside a Mylar pouch. The temperature-dependent conductivity was evaluated for both Na_3_Ga_2_Li_3_F_12_ and Na_3_Al_2_Li_3_F_12_ under dry nitrogen during heating and cooling from room temperature to 100 °C, as shown in Figures 4 and S4. The single-arc equivalent circuit and fitted values of R, Q, n, and effective capacitance at 100 °C are provided in Figure S4. Previous work on *M* = Al, Sc, and In analogs of this phase reported no observable Li-ion diffusion until 100 °C, with conductivities of ∼10^–10^ S cm^–1^ for Al and In, and ∼10^–9^ S cm^–1^ for Sc as well as activation energies of 0.95, 0.83, and 0.97 eV for Al, In, and Sc, respectively.^9^ Consistent with these prior reports, both Na_3_Al_2_Li_3_F_12_ and Na_3_Ga_2_Li_3_F_12_ show no measurable conductivity at room temperature under dry nitrogen; both yield a resolvable semicircle only above ∼100 °C, with values of 1.9 × 10^–11^ S cm^–1^ and 2.0 × 10^–11^ S cm^–1^, respectively, that are reproducible on heating and cooling. Furthermore, measurements beyond ∼100 °C were also conducted in a dry nitrogen atmosphere up to ∼300 °C. The results of these measurements on heating and cooling are shown in Figure S3a–d, with an added Arrhenius plot in Figure S3e showing the extracted activation energies for each compound, 1.27 eV for Na_3_Ga_2_Li_3_F_12_ and 0.79 eV for Na_3_Al_2_Li_3_F_12_.

**Figure 4:**
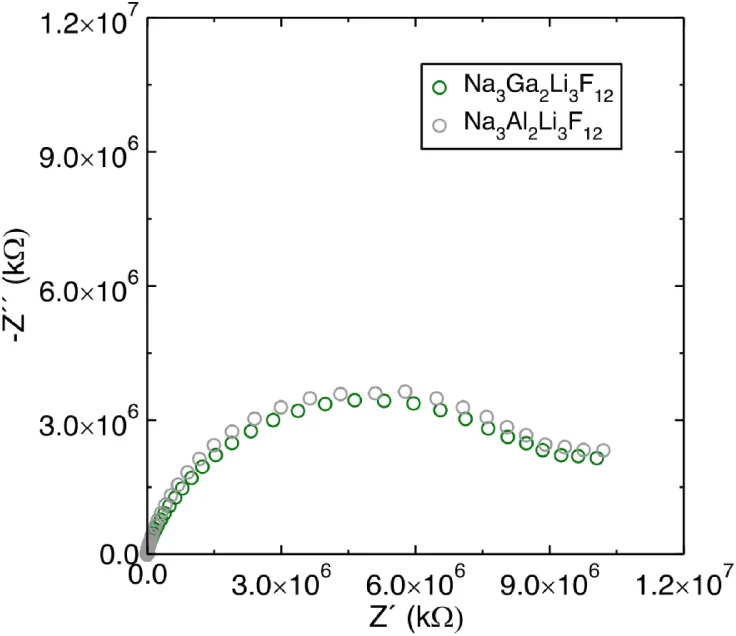
EIS Nyquist plots of Na_3_Ga_2_Li_3_F_12_ and Na_3_Al_2_Li_3_F_12_ pellets with gold-sputtered electrodes, measured at 100 °C in a sealed Mylar pouch cell with aluminum tabs.

To complement the average structure obtained from Rietveld refinement, neutron total scattering was performed to probe the local structure through pair distribution function (PDF) analysis. Starting from the synchrotron XRD structural models, the PDF data were fit over a range of 20 Å, yielding *R*_*p*_ ≈ 10% for both compounds (Figure 5; refined parameters in Supporting Tables S3–S4). The fits are generally good, reproducing both the nearest-neighbor correlations and the longer-range structure. In the Na_3_Ga_2_Li_3_F_12_ data, however, the model underfits a peak at 2.22 Å, suggesting possible gallium antisite disorder on interstitial sites within the lithium substructure. Such disorder would be significant, as non-Li cations on these sites would block the Li-ion diffusion pathways and reduce the ionic conductivity. To investigate this possibility, the data were refit with three alternative models that place gallium on (1) an empty 48*g* interstitial site, (2) the lithium 24*d* site with shared occupancy, or (3) the sodium 24*c* site with shared occupancy. Models (2) and (3) either worsened the fit or produced unphysically large displacement parameters and were discarded. Model (1) improved the low-*r* region without degrading the fit at longer distances, but the refined gallium occupancy on the 48*g* site was only ∼5%. Given this small occupancy, any blocking of the Li-ion diffusion pathway by gallium on the 48*g* interstitial would be negligible, and antisite disorder alone cannot account for the low ionic conductivity of these materials.

**Figure 5:**
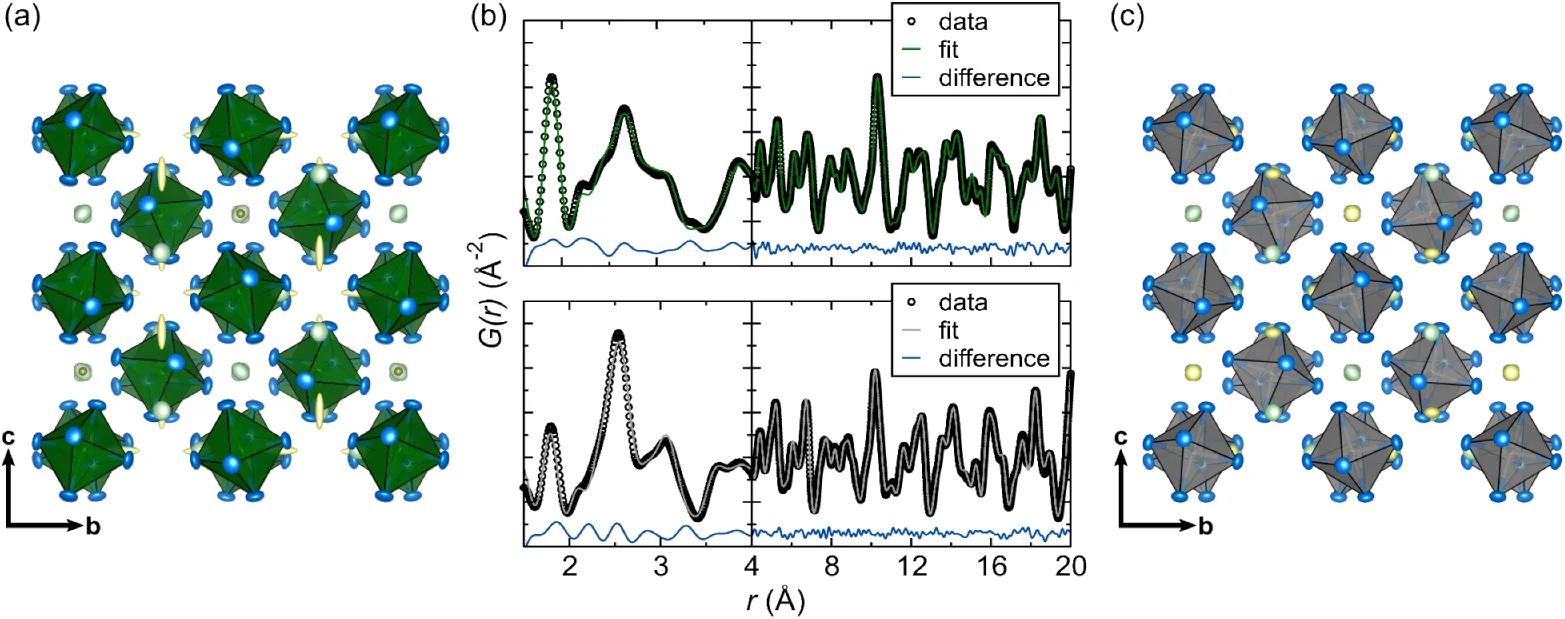
(a) Crystal structure of Na_3_Ga_2_Li_3_F_12_ and (b) neutron PDF fits to 20 Å for Na_3_Ga_2_Li_3_F_12_ (top, green) and Na_3_Al_2_Li_3_F_12_ (bottom, gray); left panels show an expanded view of the low-*r* region. (c) Crystal structure of Na_3_Al_2_Li_3_F_12_. Thermal ellipsoids in (a) and (c) are drawn from the PDF refinement. Crystal structures depicted using VESTA.^13^

As an additional probe for antisite disorder, ^71^Ga (*S* = 3/2) and ^27^Al (*S* = 5/2) MAS NMR spectra were collected. The ^71^Ga spectrum at 800 MHz (Figure 6) shows a main peak at −53 ppm with a shoulder at −56 ppm. Because ^71^Ga is a quadrupolar nucleus, a second measurement at 500 MHz was conducted to determine whether the shoulder arises from a distinct crystallographic site or from quadrupolar splitting. The increased broadening at lower field is consistent with a quadrupolar origin, confirming a single gallium environment. The ^27^Al spectrum likewise shows a single environment, with spinning sidebands and a minor peak attributable to the AlF_3_ impurity identified in the X-ray diffraction data. The observation of a single gallium environment by NMR is consistent with the low (∼5%) 48*g* occupancy from the PDF refinement, as such a minor population would fall below the NMR detection threshold. Overall, the diffraction, PDF, and NMR data show that both fluoride garnets are highly ordered.

**Figure 6:**
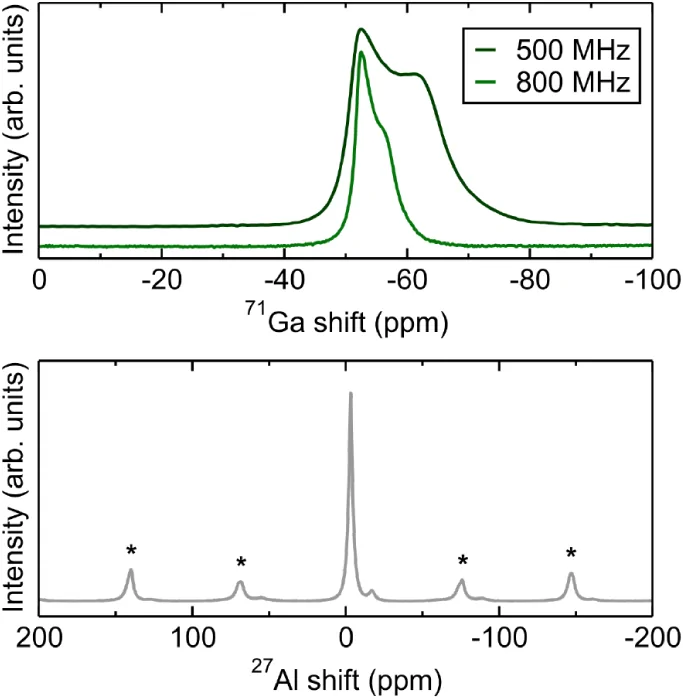
^71^Ga and ^27^Al MAS NMR spectra showing single gallium and aluminum environments in each sample. Asterisks denote spinning sidebands.

Since structural disorder cannot account for the low ionic conductivity, we used climbing-image NEB calculations to compute migration barriers for $V_{\mathrm{Li}}^{\prime }$ and ${\mathrm{Li}}_{i}^{•}$ defects in the fluoride garnet framework. The vacancy mechanism involves a single Li ion migrating between adjacent 24*d* sites via a 48*g* intermediate; the interstitial mechanism is an interstitialcy-type process in which the ${\mathrm{Li}}_{i}^{•}$ displaces a neighboring Li on a 24*d* tetrahedral site, so that two ions move cooperatively. The computed barriers are low in both materials: 0.29 eV for vacancy migration and 0.16 to 0.17 eV for the interstitialcy mechanism (Table 1, Figure 7). These barriers are significantly lower than the total experimental activation energies reported for analogous garnets with *M* = Al, Sc, and In,^9^ consistent with the experimental activation energy including a substantial contribution from defect formation.

**Table 1:** DFT-NEB Calculated Migration Barriers for Vacancy $\left(V_{\mathrm{Li}}^{\prime }\right)$ and Interstitialcy $\left({\mathrm{Li}}_{i}^{•}\right)$ Mechanisms in Na_3_Ga_2_Li_3_F_12_ and Na_3_Al_2_Li_3_F_12_

	$E_{a}\left(V_{\mathrm{Li}}^{\prime }\right)/\mathrm{eV}$	$E_{a}\left({\mathrm{Li}}_{i}^{•}\right)/\mathrm{eV}$
Na_3_Ga_2_Li_3_F_12_	0.29	0.17
Na_3_Al_2_Li_3_F_12_	0.29	0.16

**Figure 7:**
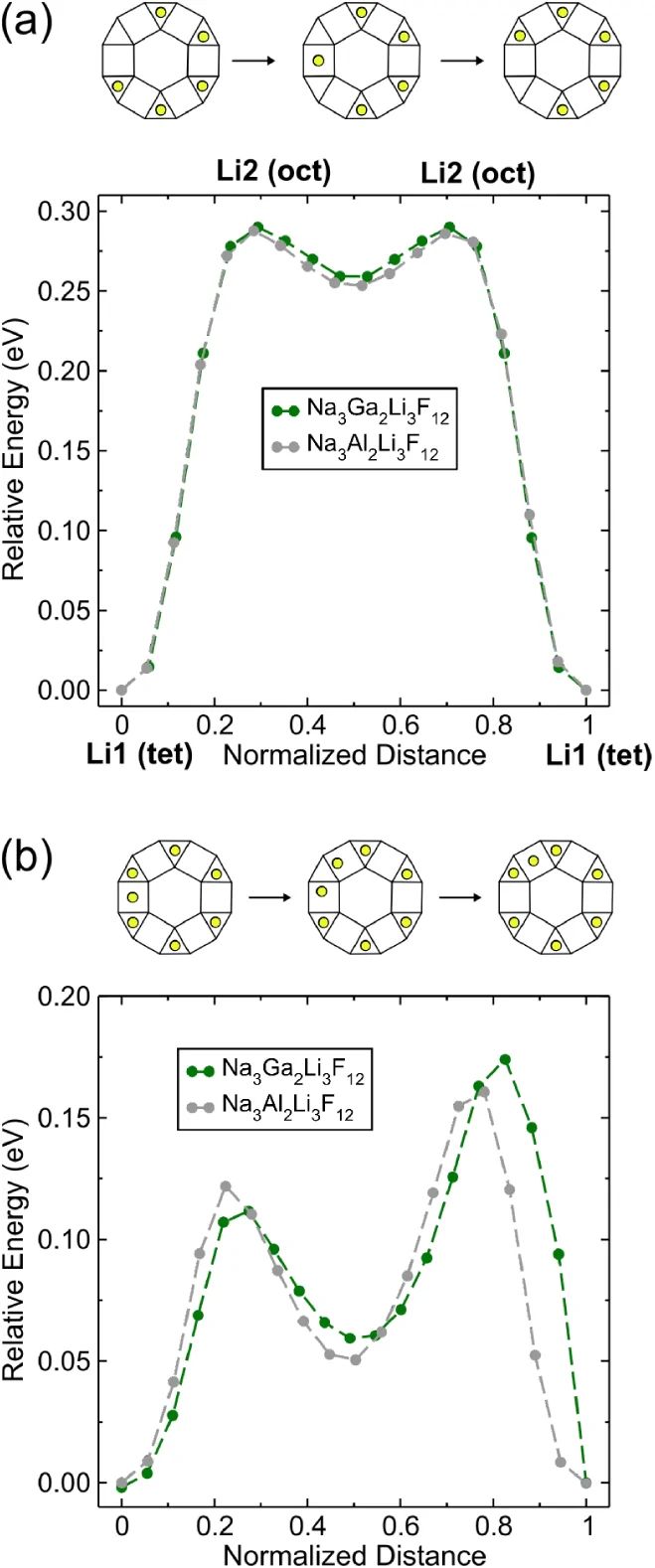
DFT-NEB migration barriers for (a) vacancy and (b) interstitialcy mechanisms in Na_3_Ga_2_Li_3_F_12_ (green) and Na_3_Al_2_Li_3_F_12_ (gray). Insets show schematic 2D projections of the diffusion pathways: triangles represent tetrahedral 24*d* sites, rectangles represent 48*g* interstitial sites. In (a), a single Li^+^ moves between adjacent 24*d* sites; in (b), two Li^+^ ions move cooperatively.

Since the calculated migration barriers are low, the poor macroscopic conductivity may instead reflect a low intrinsic concentration of mobile lithium defects. To examine this, we computed the formation energies of lithium Frenkel and lithium–fluorine Schottky defect pairs, with both constituent defects included simultaneously in the same supercell to form charge-neutral complexes.

1 $$\Delta E^{\mathrm{Frenkel}}=E\left({\mathrm{Li}}_{i}^{•}+V_{\mathrm{Li}}^{\prime }\right)-E^{\mathrm{bulk}}$$

$$\Delta E^{\mathrm{Schottky}}=E\left(V_{\mathrm{Li}}^{\prime }+V_{F}^{•}\right)-E^{\mathrm{bulk}}+\mu_{\mathrm{Li}}+\mu_{F}$$ 2

Here, Δ*E* ^Frenkel^ and Δ*E* ^Schottky^ denote the formation energies of neutral lithium Frenkel and lithium–fluorine Schottky pairs, respectively, $E\left({\mathrm{Li}}_{i}^{•}+V_{\mathrm{Li}}^{\prime }\right)$ is the energy of a supercell containing both a lithium interstitial and a lithium vacancy, $E\left(V_{\mathrm{Li}}^{\prime }+V_{F}^{•}\right)$ is the energy of a supercell containing a lithium and a fluorine vacancy, and *E* ^bulk^ is the energy of the pristine supercell. The chemical potentials μ_Li_ and μ_F_ are referenced to elemental lithium metal and molecular fluorine gas $\left(\frac{1}{2}E_{F_{2}}\right)$ , respectively. These potentials are constrained by the thermodynamic stability conditions of Na_3_Al_2_Li_3_F_12_ and Na_3_Ga_2_Li_3_F_12_, and were calculated following Kavanagh et al.^15^ The full chemical potential ranges used in the defect calculations, along with competing phase data, are provided in Tables S6–S7.

The calculated Frenkel pair formation energy is 1.91 eV for Na_3_Ga_2_Li_3_F_12_ and 2.07 eV for Na_3_Al_2_Li_3_F_12_. These values are sufficiently high that the equilibrium concentration of thermally generated Frenkel pairs will be negligibly small at temperatures relevant to solid-state battery operation. The Schottky pair formation energies are similarly high, ranging over the accessible chemical potential window from 2.01 to 2.21 eV for the Ga garnet and 2.22 to 2.39 eV for the Al garnet. Because Schottky defects are quenched in from the synthesis temperature,^16^ their equilibrium concentration is set during crystal growth rather than during battery operation; even at typical hydrothermal synthesis temperatures, however, these formation energies remain far too high to produce a significant population of vacancies. The Schottky formation energies vary by at most 0.2 eV across the entire stability field of each compound, which is far smaller than the formation energies themselves. Schottky defect concentrations will therefore be negligible under all accessible synthesis conditions.

Taken together, the high formation energies for both Frenkel and Schottky pairs predict negligible equilibrium concentrations of intrinsic Li-ion defects. The low migration barriers suggest that these defects would be mobile once formed, but their low intrinsic concentrations limit the net ionic conductivity. The comparable conductivities and activation energies reported for the Al-, Sc-, and In-containing analogs to those reported here are consistent with a common defect-limited transport mechanism, but they do not establish that the same microscopic mechanism operates throughout the fluoride-garnet family. Direct defect calculations or measurements of carrier populations in the Sc- and In-containing phases would be required to test this hypothesis. One strategy, therefore, to improve the conductivity of the fluoride garnets is aliovalent doping, which would introduce charge-compensating Li-ion defects extrinsically. We attempted to achieve this experimentally through substitution of the parent phases with Mg^2+^ and Zn^2+^ on the octahedral site; however, all attempts proved unsuccessful under hydrothermal conditions, resulting in the formation of perovskite impurities (NaMgF_3_ or NaZnF_3_). The formation of NaMgF_3_ and NaZnF_3_ is likely due to the fact that simple perovskites are thermodynamically favorable over more complex fluoride garnets under these synthetic conditions.^17–19^ Conventional solid-state and mechanochemical routes were also attempted but similarly unsuccessful at incorporating the targeted dopants.

## Conclusions

We have investigated the origin of the poor ionic conductivity in the fluoride garnets Na_3_Ga_2_Li_3_F_12_ and Na_3_Al_2_Li_3_F_12_ through a combination of structural characterization and first-principles calculations. Both compounds are highly ordered, with synchrotron X-ray diffraction, neutron pair distribution function analysis, and ^71^Ga/^27^Al MAS NMR showing no evidence for significant antisite disorder. Under controlled atmospheres, both materials exhibit intrinsic ionic conductivities of ∼2 × 10^–11^ S cm^–1^ at 100 °C; a humidity-induced enhancement observed in Na_3_Ga_2_Li_3_F_12_ is attributed to an extrinsic surface process on the basis of FTIR and TGA measurements. First-principles nudged elastic band calculations predict that the Li-ion vacancy and interstitialcy migration barriers in both materials are low (0.16 to 0.29 eV), indicating that the fluoride garnet framework does not inherently restrict Li-ion diffusion. The formation energies for lithium Frenkel (1.9 to 2.1 eV) and Schottky (2.0 to 2.4 eV) defect pairs, however, are high, meaning that the intrinsic concentrations of mobile lithium defects are negligibly small at temperatures relevant to solid-state battery operation. Attempts to introduce extrinsic defects through aliovalent substitution with Mg^2+^ and Zn^2+^ were unsuccessful under hydrothermal conditions, consistently yielding perovskite impurity phases.

Taken together, these results establish that the low conductivity of stoichiometric fluoride garnets arises from high defect-formation energies, rather than high migration barriers: the transport pathways are favorable, but cannot be exploited without a sufficient population of mobile lithium defects. Whether this limitation can be overcome remains an open question. The high thermodynamic cost of forming Frenkel and Schottky pairs suggests that equilibrium approaches to generating disorder are unlikely to succeed, and nonequilibrium synthetic routes—such as high-energy ball milling or rapid quenching from high temperature—may be needed to kinetically trap the Li-ion disorder required to activate these pathways. Alternatively, if compositions with lithium content above the stoichiometric Li_3_ level can be stabilized in the fluoride garnet framework, the resulting redistribution of lithium across the tetrahedral and interstitial sites could provide the disorder necessary for fast ion transport, as demonstrated in the stuffed oxide garnets.

## Experimental Methods

### Material Synthesis

**Caution!** *Hydrofluoric acid is toxic and corrosive and must be handled with extreme caution and the appropriate protective gear. If contact with the liquid or vapor occurs, proper treatment procedures should be followed immediately.* ^20^

Na_3_Ga_2_Li_3_F_12_ was synthesized using a hydrothermal method. Stoichiometric mixtures of the starting binary fluorides, NaF (Sigma-Aldrich, ≥99.99%), LiF (Sigma-Aldrich, 99.99%), and GaF_3_ (Thermo Scientific, 99.85%), were added to a FEP pouch (0.127 mm thick) along with 3 mL of concentrated hydrofluoric acid (Alfa Aesar, 40% v/v). The pouch was sealed with a foot-impulse sealer and placed in a 400 mL Teflon-lined stainless steel autoclave which was backfilled with 133 mL of deionized water (33% pressure vessel volume). The autoclave was heated to 150 °C over 1.5 h, held for 24 h, and subsequently cooled to room temperature over 20 h (0.1 °C min^–1^). Upon completion of the reaction the contents of the pouch were vacuum filtered and washed several times with water followed by acetone to aid in drying the product, yielding a fluffy white powder.

### Powder X-ray Diffraction

Laboratory powder X-ray diffraction (PXRD) patterns were collected on a Bruker D8 Advance diffractometer in Bragg–Brentano geometry with a Cu Kα source (λ_1_ = 1.5406 Å, λ_2_ = 1.5444 Å), equipped with a LynxEye XET detector. High-resolution synchrotron diffraction data were collected at beamline 2-1 at Stanford Synchrotron Radiation Lightsource (SSRL) using an average wavelength of 0.729213 Å. All diffraction patterns were subject to Rietveld refinements against their published crystal structures using TOPAS-Academic v6.^21^

### Neutron Total Scattering

Neutron total scattering data were collected on the NOMAD (BL-1B) beamline at the Spallation Neutron Source at Oak Ridge National Laboratory (ORNL). The pair distribution function was produced using the ADDIE software package on the ORNL analysis cluster using *Q* cutoffs of *Q*_min_ = 0.5 Å^–1^ and *Q*_max_ = 26 Å^–1^.^22^ Analysis of the pair distribution function was performed in the *PDFgui* software suite, using *Q*_broad_ and *Q*_damp_ obtained from refinement of a silicon standard and using the delta-2 parameter to account for correlated motion of the atoms.^23^

### Electrochemical Impedance Spectroscopy

To initially investigate the ionic transport properties of Na_3_Ga_2_Li_3_F_12_ and Na_3_Al_2_Li_3_F_12_, 200 mg of each powder was uniaxially pressed at 4 tons in a 10 mm die for 10 min to obtain a free-standing pellet which was subsequently pressed isostatically in a cold isostatic press (MTI) at 300 MPa for 15 min resulting in ∼1 mm thick pellets for all compounds.

Gold electrodes were sputtered onto the pellets and measured in a custom impedance cell made of Macor equipped with spring-loaded Macor plates with exposed silver wire for contact. This cell is affixed to the end of a Macor rod inside a glass shroud designed to fit inside a tube furnace. Measurements were conducted under flowing argon at ∼10 mL min^–1^ between 25 and 75 °C inside a Thermo Scientific Lindberg/Blue M Mini-Mite tube furnace. Spectra were collected in the frequency range of 10 mHz to 1 MHz on a Solartron 1260A Impedance Analyzer.

Further measurements to investigate the high temperature behavior of the materials were conducted in the Zeier lab at the University of Münster. Pellets of Na_3_Ga_2_Li_3_F_12_ and Na_3_Al_2_Li_3_F_12_ were sputtered with 150 nm of Au and affixed with aluminum metal terminals and sealed within a Mylar pouch cell. Measurements were conducted in a ZGS active sample cell from Novocontrol with 100 mV amplitude with varying frequency from 10 MHz to 10 mHz and varying temperature from 25 to 100 °C.

Time-dependent humidity measurements were also conducted at the University of Münster in a custom setup involving the flow of dry nitrogen through two saturated solutions of Ca­(NO_3_)_2_ to create a humid atmosphere. The setup is equipped with a hygrometer which measured ∼52% relative humidity and a thermocouple that measured 22 °C for the room temperature measurement. EIS conditions mirrored those used in the ZGS sample cell, 100 mV amplitude sweeping frequency from 10 MHz to 10 mHz.

### Scanning Electron Microscopy

Scanning electron microscopy images were collected on a Thermo Fisher Scientific Apreo 2 SEM. Imaging was conducted at 5 kV accelerating voltage at a working distance of 10 mm in OptiPlan mode utilizing the T2 detector. Samples were pressed onto double-sided carbon tape (Ted Pella) and sputter coated with Pt/Pd for 30 s before imaging to prevent charging.

### Magic-Angle-Spinning NMR

^27^Al and ^71^Ga magic-angle-spinning (MAS) NMR experiments were carried out with a Bruker Advance III 800 MHz spectrometer. A home-built low-E 3.2 mm MAS ^1^H-X-Y probe was tuned to 208.489 and 244.103 MHz for the measurements of ^27^Al and ^71^Ga, respectively. Pencil rotors (3.2 mm) containing the powdered samples were spun at 15 kHz. A single-pulse (π/2) sequence was used for ^27^Al measurements, and a rotor-synchronized spin–echo sequence (π/2−π) was used for ^71^Ga measurements. π/2 pulse lengths of 3.25 and 3.50 μs were used for ^27^Al and ^71^Ga, respectively. ^27^Al shifts were referenced to solid AlF_3_ at −17 ppm. ^71^Ga shifts were referenced to 1 M Ga(NO_3_)_3_ solution at 0 ppm.

^71^Ga MAS NMR experiments were also carried out with a Bruker Avance III 500 MHz spectrometer to confirm quadrupolar coupling effects. A Bruker Micro 2.5 mm probe was tuned to 152.523 MHz. Samples were packed into 2.5 mm zirconia rotors and spun at 25 kHz. A rotor-synchronized spin–echo sequence (π/2−π) was used with a π/2 pulse length of 5.0 μs. ^71^Ga shifts were referenced to 1 M Ga­(NO_3_)_3_ solution at 0 ppm.

### DFT Calculations

Density functional theory (DFT) calculations were performed using the Vienna *Ab initio* Simulation Package (VASP) with the Projector Augmented Wave (PAW) method.^24–27^ The exchange–correlation energy was treated using the PBEsol generalized gradient approximation (GGA) functional.^28^

A plane-wave energy cutoff of 500 eV was used throughout, and increased by 30% for calculations involving variable cell relaxations. Reciprocal space was sampled using a Γ-centered Monkhorst–Pack *k*-point mesh with a density of 0.36 Å^–1^ for the garnet phases. For all competing phases, *k*-point grids were converged individually to ensure the total energy was accurate to within 1 meV per atom.

Electronic self-consistency was deemed achieved when the total energy difference between successive steps fell below 1 × 10^–6^ eV. Structural relaxations were considered converged when the forces on all atoms were less than 0.005 eV Å^–1^. For the nudged elastic band (NEB) calculations, a looser force threshold of 0.01 eV Å^–1^ was applied. Migration barriers were calculated using the climbing-image NEB method as implemented by Henkelman et al.^29^ Symmetry-distinct defect-pair configurations in the 80-atom garnet primitive cell were enumerated using bsym,^30^ and from these the configurations with the largest defect-pair separations were selected for calculation.

## Supplementary Material



## Notes

Powder X-ray diffraction Rietveld refinement results; neutron pair distribution function fitting results; variable-temperature electrochemical impedance spectroscopy; thermogravimetric analysis and differential scanning calorimetry; ATR-FTIR spectra; chemical potential limits for computational studies (PDF)
