# Supplementary material for: High Defect Formation Energies Limit Ionic Conductivity in Na3Ga2Li3F12 and Na3Al2Li3F12
Source: Chem Mater. 2026 Oct 23;38(21):10973–80. doi: 10.1021/acs.chemmater.6c01542 (PMC13757702; doi:10.1021/acs.chemmater.6c01542)
Supplement: Supplementary file 1 [file cm6c01542_si_001.pdf]

High Defect Formation Energies Limit Ionic Conductivity in $Na_3Ga_2Li_3F_{12}$ and $Na_3Al_2Li_3F_{12}$

Michael J. Brady,[†] Hauke Glück,[‡] Jessica L. Andrews,[†] Erica Truong,[¶] Alexander G. Squires,[§,‖] Ifeoluwa P. Oyekunle,[¶] Rachel C. Monger,[⊥] Wolfgang G. Zeier,[‡,#] Yan-Yan Hu,[¶,@] David O. Scanlon,[§,‖] Benjamin J. Morgan,[△] and Brent C. Melot[*,∇]

†*Department of Chemistry, University of Southern California, Los Angeles, California 90089, USA*

‡*Institute for Inorganic and Analytical Chemistry, University of Münster, Corrensstrasse 30, D-48149 Münster, Germany*

¶*Department of Chemistry and Biochemistry, Florida State University, Tallahassee, FL 32306, USA*

§*School of Chemistry, University of Birmingham, Birmingham, B15 2TT UK, United Kingdom*

‖*The Faraday Institution, Quad One, Harwell Science and Innovation Campus, Didcot, OX11 0RA, U.K.*

⊥*Department of Chemical Engineering and Materials Science, University of Southern California, Los Angeles, CA 90089, USA*

#*Institute of Energy Materials and Devices (IMD), IMD-4: Helmholtz-Institut Münster, Forschungszentrum Jülich, Corrensstraße 46, D-48149 Münster, Germany*

@*Center for Interdisciplinary Magnetic Resonance, National High Magnetic Field Laboratory, Tallahassee, FL 32306, USA*

△*Department of Chemistry, University of Bath, Claverton Down, Bath BA2 7AY, U.K.*

∇*Departments of Chemistry and Materials, Binghamton University, Binghamton, New York 13850-6000, USA*

E-mail: bmelot@binghamton.edu

Powder X-Ray Diffraction Rietveld Refinement Results

Table S1: Rietveld refinement of $Na_3Ga_2Li_3F_{12}$ against synchrotron powder X-ray diffraction data.

**Space Group**	$\boldsymbol{a}$ **(Å)**				$\boldsymbol{R_{wp}}$	
$Ia\bar{3}d$	12.30026(2)				1.7	
**Atom**	**Wyckoff**	$\boldsymbol{X}$	$\boldsymbol{Y}$	$\boldsymbol{Z}$	**Occ.**	$\boldsymbol{U_{iso}}$
Na	24*c*	1/8	0	1/4	1.0	0.02059
Ga	16*a*	0	0	0	1.0	0.00909
Li	24*d*	3/8	0	1/4	1.0	0.00756
F	96*h*	−0.02918(4)	0.04519(4)	0.14252(4)	1.0	0.01421

Table S2: Rietveld refinement of $Na_3Al_2Li_3F_{12}$ against synchrotron powder X-ray diffraction data.

**Space Group**	$\boldsymbol{a}$ **(Å)**				$\boldsymbol{R_{wp}}$	
$Ia\bar{3}d$	12.1262(2)				3.9	
**Atom**	**Wyckoff**	$\boldsymbol{X}$	$\boldsymbol{Y}$	$\boldsymbol{Z}$	**Occ.**	$\boldsymbol{U_{iso}}$
Na	24*c*	1/8	0	1/4	1.0	0.01883
Al	16*a*	0	0	0	1.0	0.00861
Li	24*d*	3/8	0	1/4	1.0	0.01408
F	96*h*	−0.028778(19)	0.04236(2)	0.139464(19)	1.0	0.01391

Neutron Pair Distribution Function Fitting Results

Table S3: Neutron pair distribution function refinement of $Na_3Ga_2Li_3F_{12}$.

**Space Group**	***a*** **(Å)**				***R_p***	
$Ia\bar{3}d$	12.28280				11.4	
**Atom**	**Wyckoff**	***X***	***Y***	***Z***	**Occ.**	
Na	24*c*	1/8	0	1/4	1.0	
Ga	16*a*	0	0	0	1.0	
Li	24*d*	3/8	0	1/4	1.0	
F	96*h*	0.970299	0.045273	0.143	1.0	
**Atom**	U_{11}	U_{22}	U_{33}	U_{12}	U_{13}	U_{23}
Na	0.019460	0.016290	0.016290	0	0	0.004120
Ga	0.006980	0.006980	0.006980	0.001110	0.001110	0.001110
Li	0.061660	0.003800	0.003800	0	0	0
F	0.015400	0.016960	0.005760	0.001030	0.002370	−0.002120

Table S4: Neutron pair distribution function refinement of $Na_3Al_2Li_3F_{12}$.

**Space Group**	***a*** **(Å)**				***R_p***	
$Ia\bar{3}d$	12.11280				12.1	
**Atom**	**Wyckoff**	***X***	***Y***	***Z***	**Occ.**	
Na	24*c*	1/8	0	1/4	1.0	
Al	16*a*	0	0	0	1.0	
Li	24*d*	3/8	0	1/4	1.0	
F	96*h*	0.970461	0.042588	0.140204	1.0	
**Atom**	U_{11}	U_{22}	U_{33}	U_{12}	U_{13}	U_{23}
Na	0.014790	0.013300	0.013300	0	0	0.003390
Al	0.005640	0.005640	0.005640	0.000040	0.000040	0.000040
Li	0.006760	0.012480	0.012480	0	0	0
F	0.013170	0.013750	0.006590	0.000410	0.001850	−0.000830

Table S5: Neutron pair distribution function refinement of a modified $Na_3Ga_2Li_3F_{12}$ structure with Ga on the 48g interstitial site.

**Space Group**	$\boldsymbol{a}$ **(Å)**				$\boldsymbol{R_p}$	
$Ia\bar{3}d$	12.2827				11.4	
**Atom**	**Wyckoff**	$\boldsymbol{X}$	$\boldsymbol{Y}$	$\boldsymbol{Z}$	**Occ.**	
Na	24c	1/8	0	1/4	1.0	
Ga1	16a	0	0	0	0.9566	
Ga2	48g	1/8	0.308868	0.941132	0.0434	
Li	24d	3/8	0	1/4	1.0	
F	96h	0.970290	0.045310	0.143895	1.0	
**Atom**	$\boldsymbol{U_{11}}$	$\boldsymbol{U_{22}}$	$\boldsymbol{U_{33}}$	$\boldsymbol{U_{12}}$	$\boldsymbol{U_{13}}$	$\boldsymbol{U_{23}}$
Na	0.019460	0.016290	0.016290	0	0	0.004120
Ga1	0.006980	0.006980	0.006980	0.001110	0.001110	0.001110
Ga2	0.025845	0.030716	0.030716	0.005637	0.005637	0.005247
Li	0.061660	0.003800	0.003800	0	0	0
F	0.015400	0.016960	0.005760	0.001030	0.002370	−0.002120

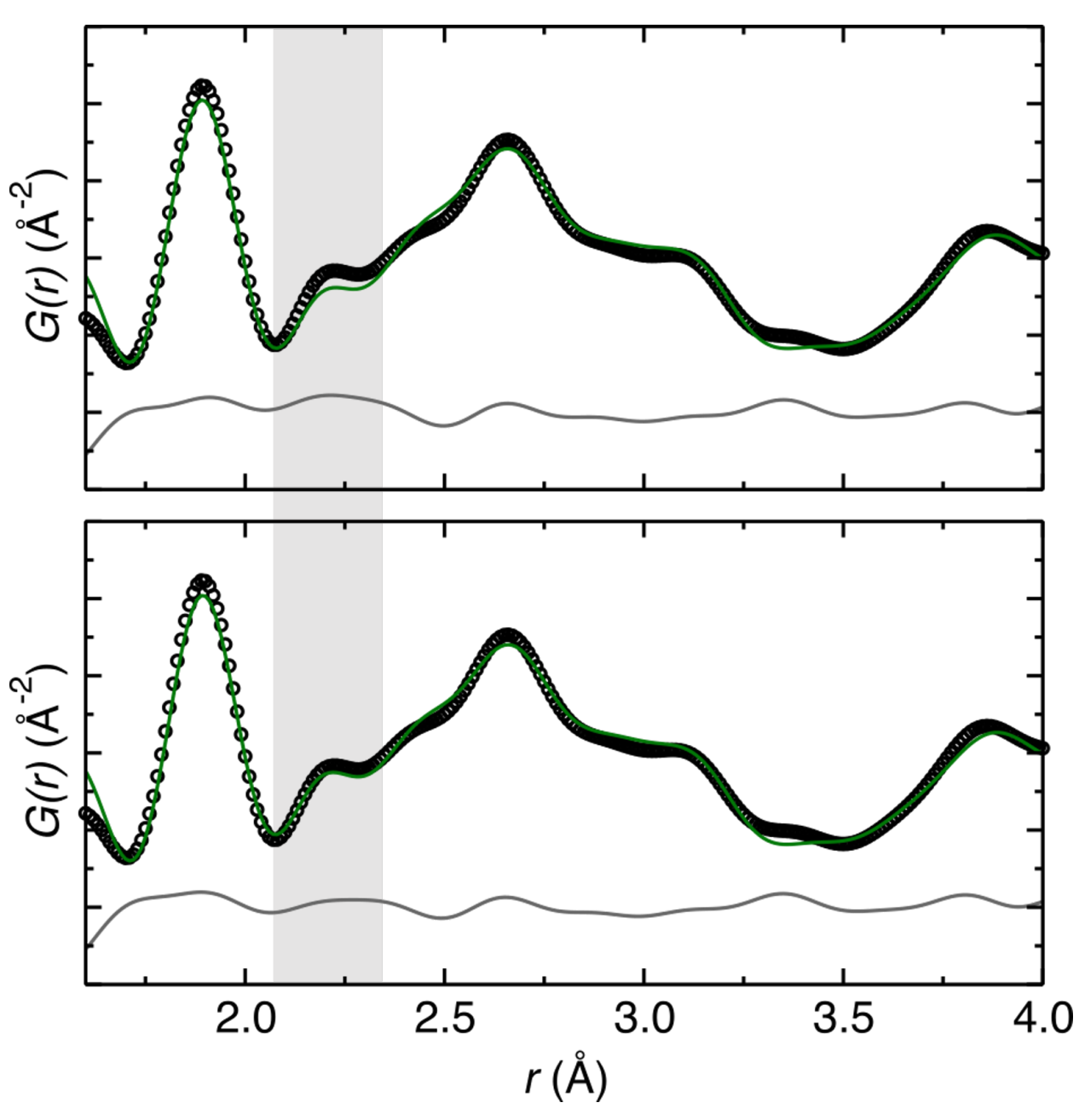

Figure S1: Enlarged view of the neutron PDF fit for the parent garnet structure (top) and the modified model with Ga occupancy on the 48g interstitial site (bottom), showing improved agreement in the low-r region near 2.22 Å.

Electrochemical Impedance Spectroscopy

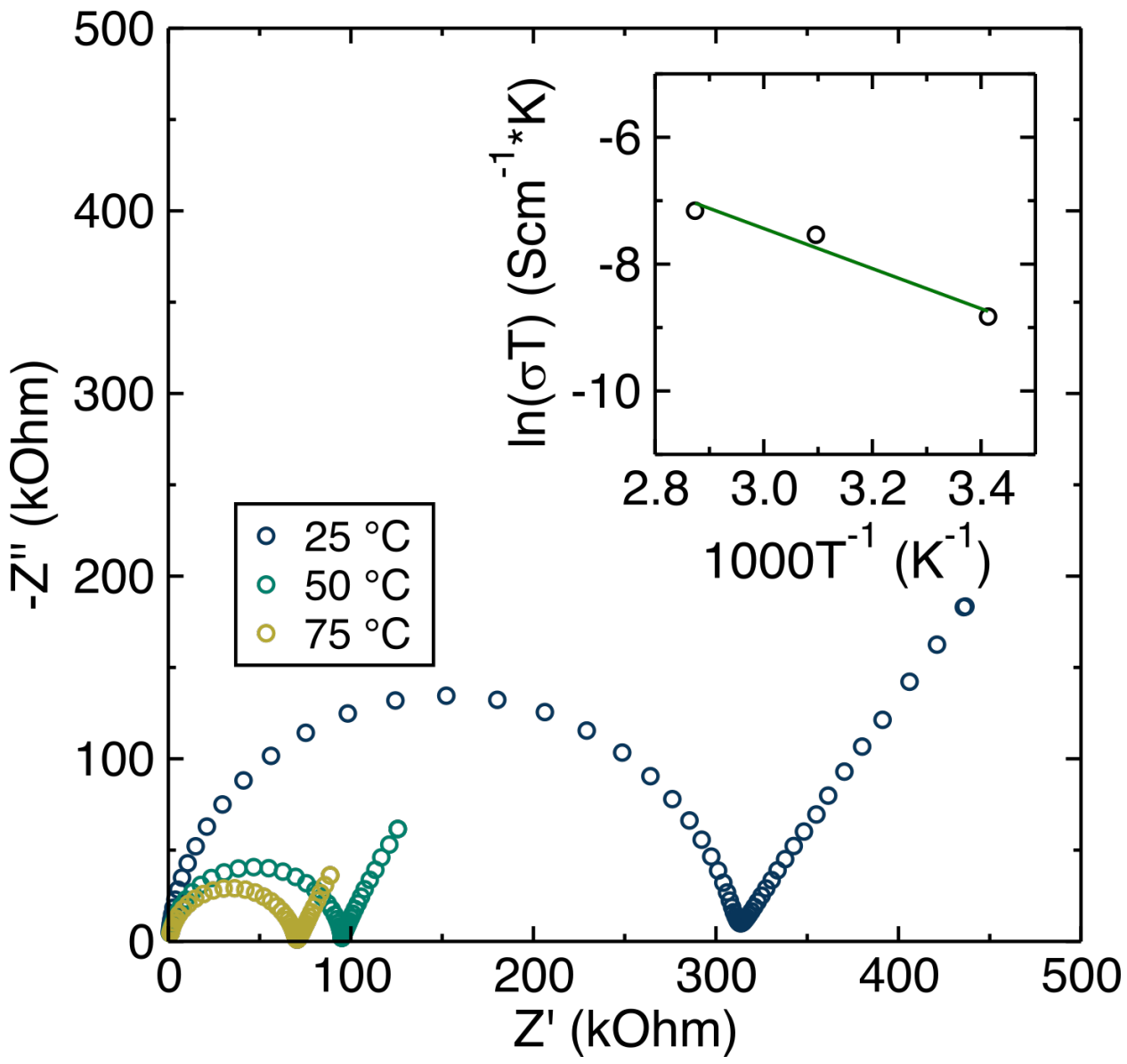

Figure S2: EIS measurements of $Na_3Ga_2Li_3F_{12}$ with gold-sputtered electrodes measured in air. The apparent high conductivity is attributed to surface-adsorbed protons rather than bulk Li-ion transport (see main text).

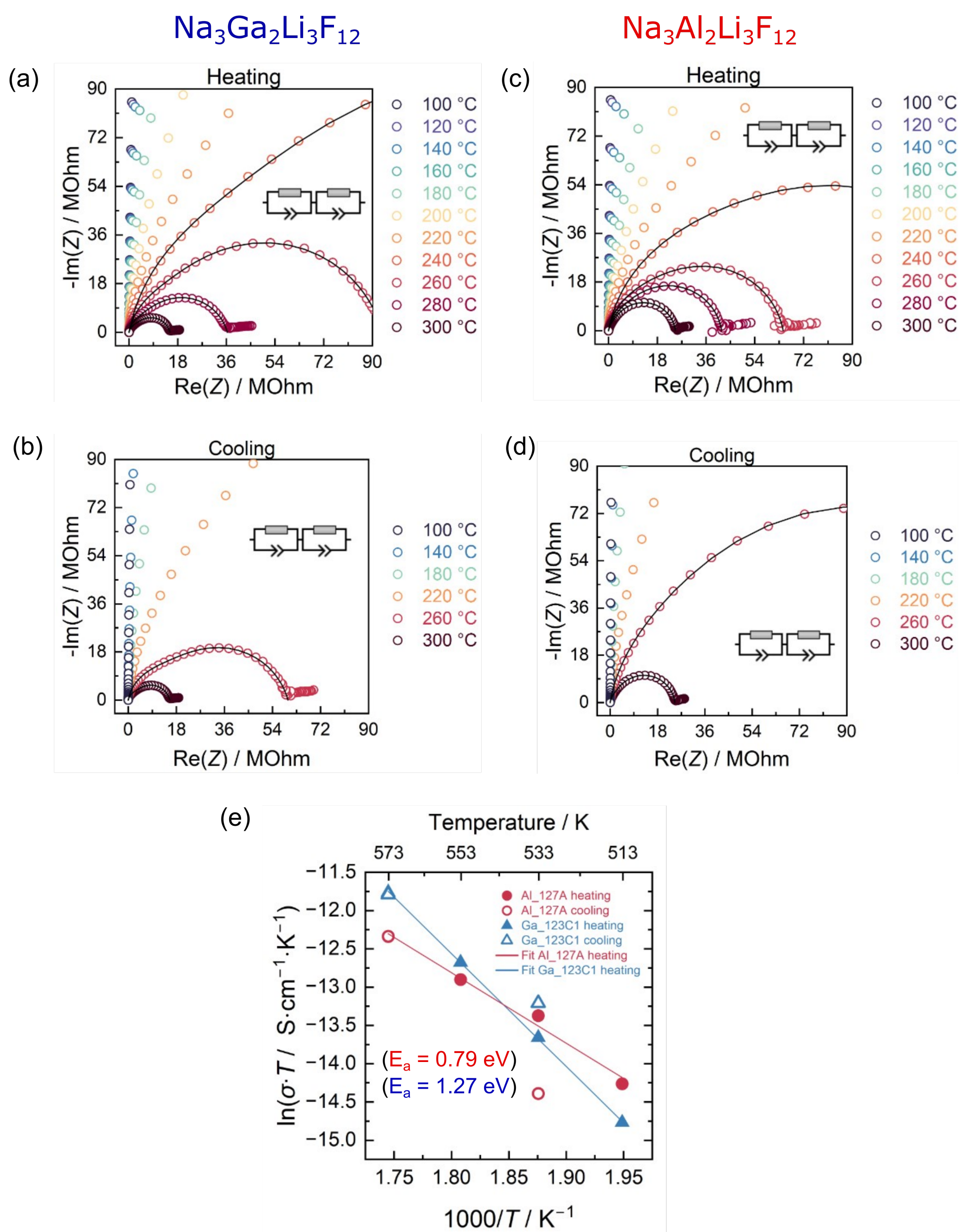

Figure S3: (a-b) Temperature-dependent EIS of $Na_3Ga_2Li_3F_{12}$ and (c-d) $Na_3Al_2Li_3F_{12}$ measured in a nitrogen atmosphere with Pt Gauze contacts on heating and cooling, respectively, up to 300 °C. Inset shows the equivalent circuit used to fit these data and extract a resistance value for conductivity calculation. (e) Arrhenius plot showing the extracted activation energy of the two compounds.

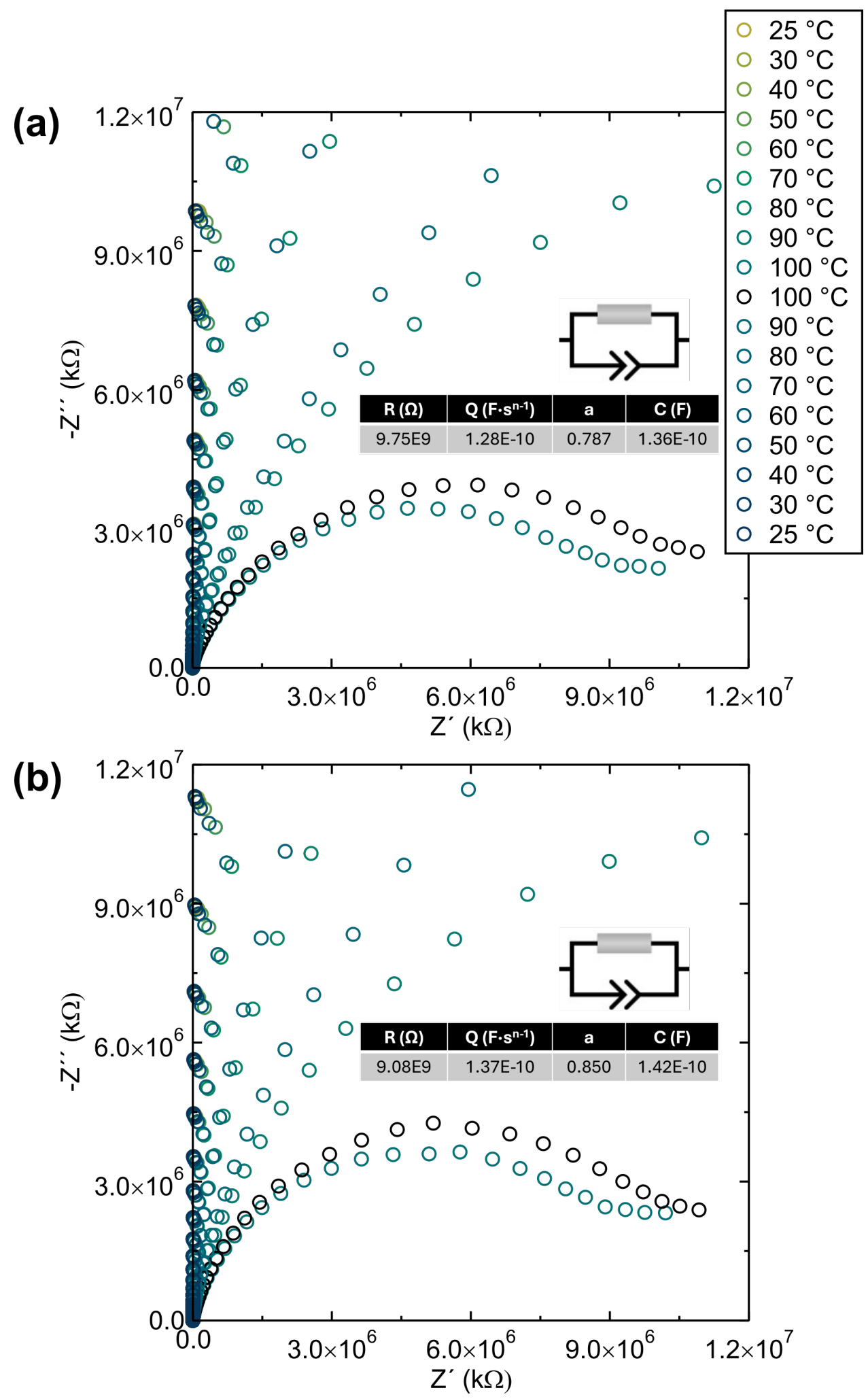

Figure S4: Temperature-dependent EIS of (a)$Na_3Ga_2Li_3F_{12}$ and (b)$Na_3Al_2Li_3F_{12}$ measured in a Mylar pouch cell on heating and cooling up to 100 °C. Inset tables show the fitting parameters for the 100 °C curves.

Thermogravimetric Analysis and Differential Scanning Calorimetry

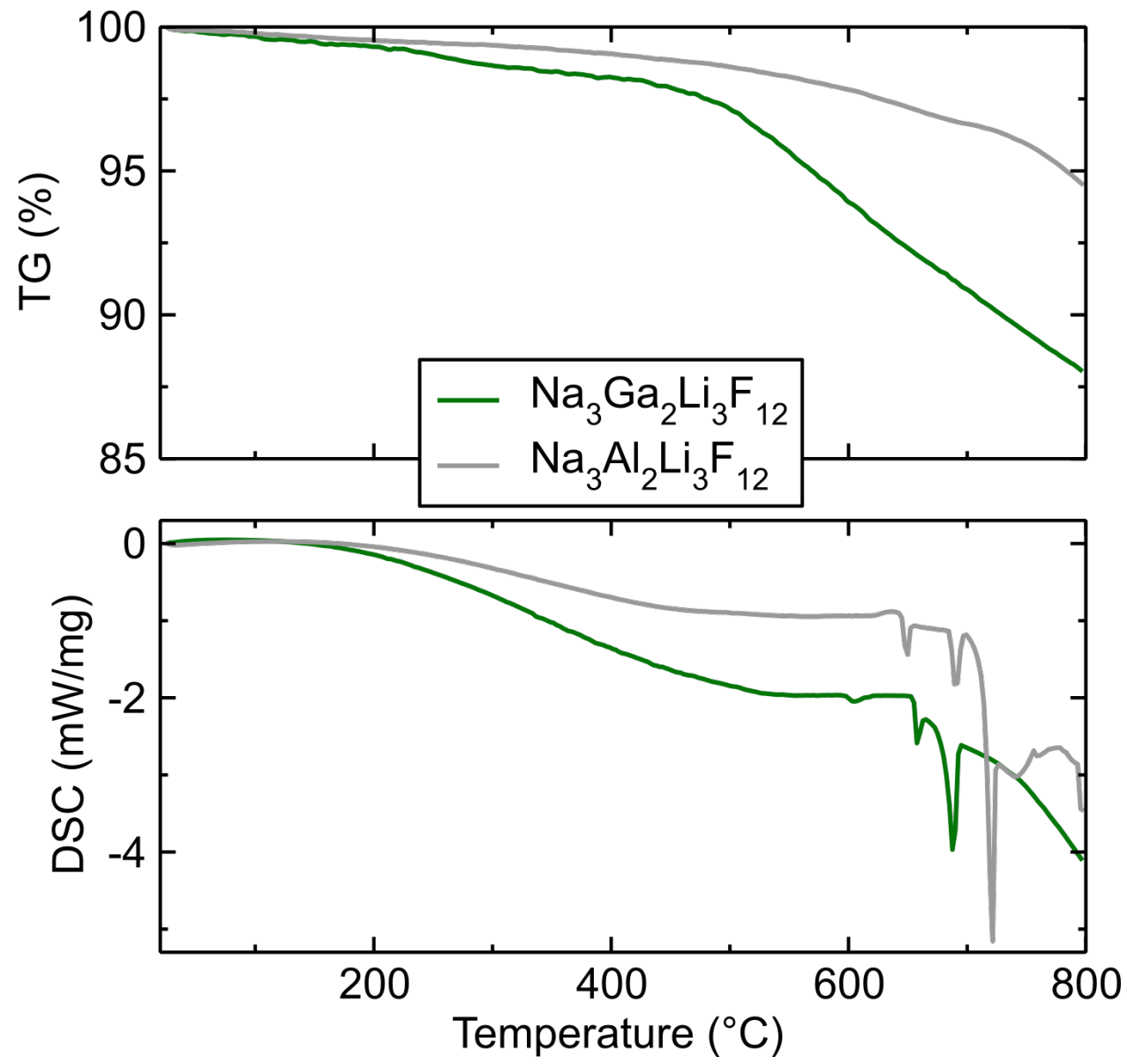

Figure S5: TGA and DSC traces of $Na_3Ga_2Li_3F_{12}$ and $Na_3Al_2Li_3F_{12}$ measured from room temperature to 800 °C. Neither compound shows significant weight loss below 500 °C; the first DSC feature appears above 600 °C for $Na_3Ga_2Li_3F_{12}$ and at a slightly higher temperature for $Na_3Al_2Li_3F_{12}$.

Attenuated Total Reflectance Fourier-Transform Infrared Spectroscopy (ATR-FTIR)

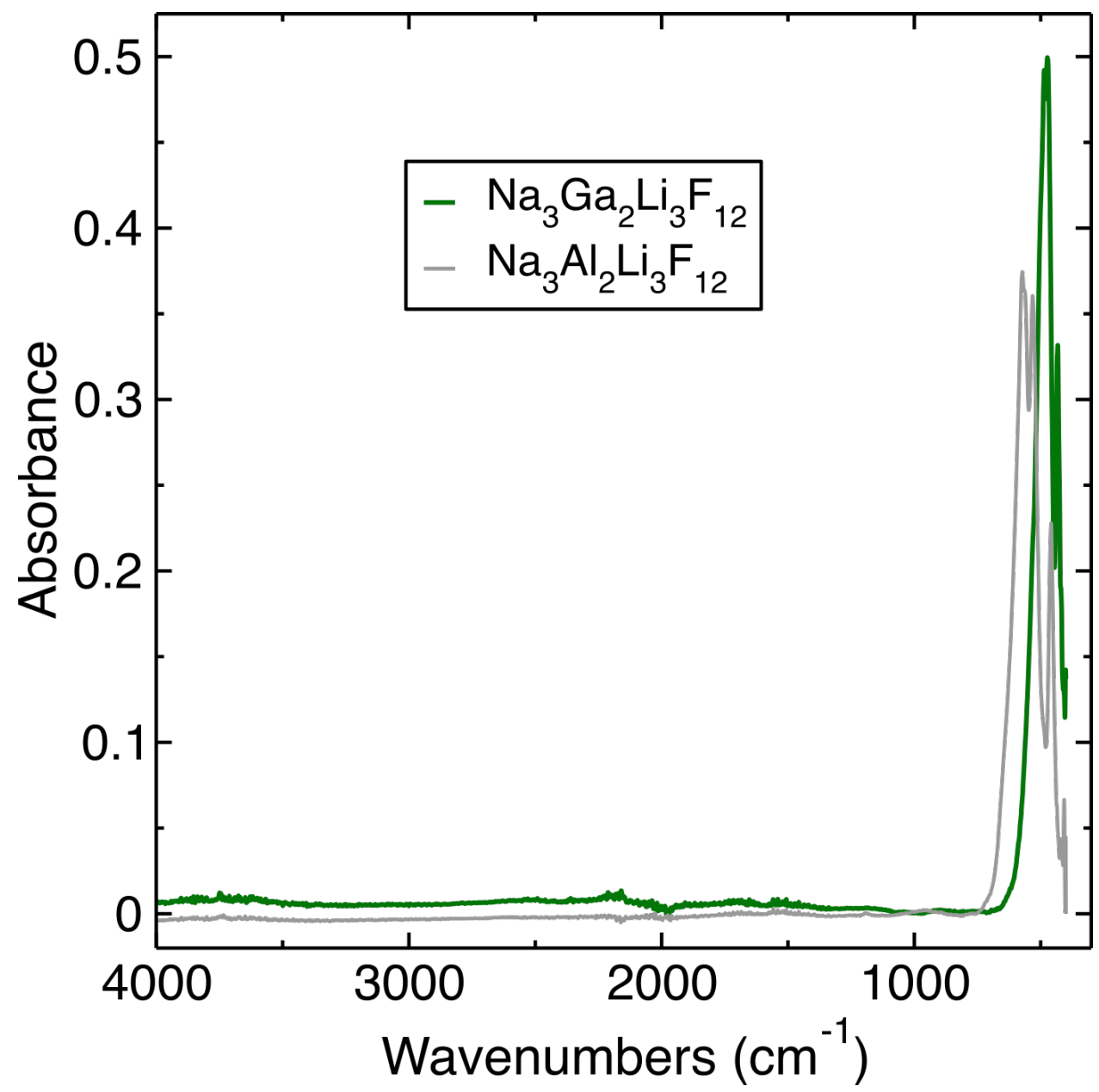

Figure S6: ATR-FTIR spectra of $Na_3Ga_2Li_3F_{12}$ and $Na_3Al_2Li_3F_{12}$ showing no absorption features in the O–H stretching region near 3500 cm^{-1}, indicating negligible water or hydroxide content.

Chemical Potential Limits

Table S6: Chemical potential limits (in eV) for $Na_3Li_3Al_2F_{12}$ in equilibrium with competing phases.

Competing Phases	μ_{Na}	μ_{Li}	μ_{Al}	μ_F
$Na_3Li_3Al_2F_{12}$–Na_3AlF_6–LiF–Al	−1.95	−2.95	−4.09	−6.91
$Na_3Li_3Al_2F_{12}$–Na_3AlF_6–LiF–F_2	−6.93	−7.93	−19.02	−1.93
Li_3AlF_6–$Na_3Li_3Al_2F_{12}$–Al–AlF_3	−2.45	−3.24	−4.09	−6.71
Li_3AlF_6–$Na_3Li_3Al_2F_{12}$–LiF–Al	−2.27	−3.06	−4.09	−6.80
Li_3AlF_6–$Na_3Li_3Al_2F_{12}$–F_2–AlF_3	−7.23	−8.02	−18.43	−1.93
Li_3AlF_6–$Na_3Li_3Al_2F_{12}$–LiF–F_2	−7.14	−7.93	−18.70	−1.93
$NaAlF_4$–$Na_3Li_3Al_2F_{12}$–Al–AlF_3	−2.39	−3.30	−4.09	−6.71
$NaAlF_4$–$Na_3Li_3Al_2F_{12}$–F_2–AlF_3	−7.17	−8.08	−18.43	−1.93
$Na_5Al_3F_{14}$–$Na_3Li_3Al_2F_{12}$–Na_3AlF_6–Al	−2.28	−3.28	−4.09	−6.74
$Na_5Al_3F_{14}$–$NaAlF_4$–$Na_3Li_3Al_2F_{12}$–Al	−2.33	−3.30	−4.09	−6.73
$Na_5Al_3F_{14}$–$Na_3Li_3Al_2F_{12}$–Na_3AlF_6–F_2	−7.09	−8.10	−18.53	−1.93
$Na_5Al_3F_{14}$–$NaAlF_4$–$Na_3Li_3Al_2F_{12}$–F_2	−7.12	−8.10	−18.48	−1.93

Table S7: Chemical potential limits (in eV) for $Na_3Li_3Ga_2F_{12}$ in equilibrium with competing phases.

Competing Phases	μ_{Na}	μ_{Li}	μ_{Ga}	μ_F
$Na_3Li_3Ga_2F_{12}$–NaF–LiF–F_2	−6.84	−7.93	−14.72	−1.93
$Na_3Li_3Ga_2F_{12}$–NaF–LiF–Ga	−3.05	−4.14	−3.35	−5.72
Li_3GaF_6–$Na_3Li_3Ga_2F_{12}$–F_2–GaF_3	−7.28	−8.05	−13.89	−1.93
Li_3GaF_6–$Na_3Li_3Ga_2F_{12}$–LiF–F_2	−7.15	−7.93	−14.26	−1.93
Li_3GaF_6–$Na_3Li_3Ga_2F_{12}$–Ga–GaF_3	−3.76	−4.54	−3.35	−5.44
Li_3GaF_6–$Na_3Li_3Ga_2F_{12}$–LiF–Ga	−3.52	−4.30	−3.35	−5.57
$Na_5Ga_3F_{14}$–$Na_3Li_3Ga_2F_{12}$–F_2–GaF_3	−7.20	−8.13	−13.89	−1.93
$Na_5Ga_3F_{14}$–$Na_3Li_3Ga_2F_{12}$–NaF–F_2	−6.84	−8.09	−14.48	−1.93
$Na_5Ga_3F_{14}$–$Na_3Li_3Ga_2F_{12}$–Ga–GaF_3	−3.69	−4.62	−3.35	−5.44
$Na_5Ga_3F_{14}$–$Na_3Li_3Ga_2F_{12}$–NaF–Ga	−3.13	−4.38	−3.35	−5.64
